# New Viewpoint in Exaggerated Increase of PtiO_2_ With Normobaric Hyperoxygenation and Reasons to Limit Oxygen Use in Neurotrauma Patients

**DOI:** 10.3389/fmed.2018.00119

**Published:** 2018-05-22

**Authors:** Gurgen Harutyunyan, Garnik Harutyunyan, Gagik Mkhoyan

**Affiliations:** ^1^Emergency, Hospital 9 de Octubre, Valencia, Spain; ^2^Faculty of Pharmacy, Universitat De València, Valencia, Spain; ^3^Anesthesiology and Intensive Care, Erebouni Medical Center, Yerevan, Armenia

**Keywords:** normobaric hyperoxia, PtiO_2_, metabolism, brain trauma, blood-buffering capacity

Multiple studies have shown that some of cerebral metabolic abnormalities occurring after traumatic brain injuries (TBI) are not of ischemic origin (1–4). The described pattern is called “metabolic crisis without brain ischemia” (5), and is associated with poor outcome within 6 months after trauma (6–8). Nevertheless, targeting adequate cerebral blood flow (CBF) and oxygen delivery (DO_2_) remains the cornerstone in the clinical management of TBI, especially in the early phase. Surprisingly, DO_2_ targeted therapy based on an escalation of dissolved O_2_, has been shown positive outcome in a few cases only, although it causes a significant increase in O_2_ pressure in cerebral tissue (PtiO_2_). High inspired O_2_ fraction or normobaric hyperoxia (NH) leads to a dramatic elevation of PtiO_2_ to “arterial” levels of 147 ± 36 mmHg (9). This daily and well-known phenomenon so far does not have definitive explanation and differs from the classical notions: oxyhemoglobin saturation (SO_2_) must remain nearly 100% over a wide range of O_2_ partial pressures (PO_2_) greater than 80 mmHg (10). NH induces a negligible increase in the amount of arterial O_2_ content and DO_2_, whereas it is associated with an important augmentation of PtiO_2_, without significant change in cerebral metabolic rate of O_2_ consumption (CMRO_2_) (11–14). Furthermore, studies using positron emission tomography, magnetic resonance imaging (MRI), and near-infrared spectroscopy showed an active O_2_ extraction fraction (OEF) in the non-necrotic tissue during NH (9, 15–18) with negligible increase of regional SO_2_ (rSO_2_) at 2.8 ± 1.82% (9) or without changes in rSO_2_ in tissue with intact autoregulation (16). The data of OEF at 0.56 ± 0.06 in reversible tissue, which although reduced from viable tissue to infarction (19), discards the possibility of a luxury perfusión in these cases. In general, NH causes an insignificant increase or no in rSO_2_ with an exaggerated elevation of PtiO_2_, which is equal to or less than end capillary PO_2_ (20). Thus, with NH the PO_2_ of end capillary venous blood reaches to “arterial” levels. Therefore, the exaggerated increase of PtiO_2_ with a negligible increase of rSO_2_ is incompatible with the classical sigmoidal form of the oxyhemoglobin dissociation curve (ODC). Hereby, the circulated hypotheses of mitochondrial dysfunction as a contributor (21) to high PtiO_2_ and the loss of O_2_ homeostatic mechanisms in the injured tissue during NH (22) requires an alternative explanation based on the conformational change of hemoglobin (Hb) quaternary structure (Max Perutz—the Nobel prize in chemistry 1962). This change produces a significant decrease in Hb–O_2_ affinity, considerable Hb buffering capacity augmentation, and convert the sigmoidal form of ODC to hyperbola. In this commentary, we explain the mechanisms underlying the exaggerated rise in PtiO_2_ with NH and consider the factors that limiting oxygen use in the damaged cerebral tissue.

## Basic Biochemistry

Hemoglobin plays an important role in the transport of CO_2_ by the blood. In the brain tissue with a respiratory quotient (RQ) of 1, metabolism of 1 mol of O_2_ results in 1 mol of CO_2_. Also, 1 mol of CO_2_ in the erythrocyte generates 1 mol of protons (H^+^) by two mechanisms:

$${\text{CO}}_{\text{2}}+{\text{ H}}_{\text{2}}\text{O }↔{\text{ HCO}}_{\text{3}}{\;}^{-}+{\text{ H}}^{+}*$$

$${\text{CO}}_{\text{2}}+{\text{ Hb-NH}}_{\text{2}}↔{\text{ Hb-NH-COO}}^{-}+{\text{ H}}^{+}$$

(*This reaction is catalyzed by carbonic anhydrase ranging between 10^4^ and 10^6^ reactions per second (23); therefore, the participation of other buffers of blood in CO_2_ buffering is not discussed).

The H^+^ uptake by Hb facilitates CO_2_ transport by stimulating bicarbonate formation: erythrocyte membranes do have a rapid anion exchange protein (capnoporin), which exchanges ${\text{HCO}}_{\text{3}}^{-}$ for Cl^−^ at a ratio of 1:1. The H^+^ released by carbamate formation is taken up by the Hb as well. Thus, Hb remains the main buffer for protons (i.e., for CO_2_), but its capacity is biochemically limited (24):

(1) $$\text{Hb }{\left({\text{O}}_{2}\right)}_{k}-{\text{ O}}_{\text{2}}↔\text{ Hb }{\left({\text{O}}_{2}\right)}_{k-1}+\;0.{\text{6H}}^{+}$$

where *k* = 1, 2, 3 or 4; 0.6 is the Haldane coefficient (the amount of the oxygen-linked H^+^ binding of Hb).

Thus, the release of 1 mol of O_2_ will allow the Hb to bind a maximum 0.6 mol of H^+^ or the Hb buffering capacity is lost in the brain tissue when OEF exceeded 60% (i.e., full saturation of Hb with H^+^) and CO_2_ begins to accumulate intensively in the brain tissue, provoking important acidosis. The imbalance of Hb capacities as a H^+^ buffer and as an O_2_ transporter in this setting (i.e., Eq. 1) makes the buffering capacity of Hb more important for brain cell survival to maintain a stable pH than its O_2_ carrier function. With the constant CMRO_2_, unchanged capillary permeability, temperature, pH, and DO_2_, the PtiO_2_ generated depends only on the Hb–O_2_ affinity, which decreases when Hb is saturated with negative allosteric effectors [2,3-diphosphoglycerate (2,3 DPG), CO_2_, H^+^, Cl^−^] (24).

The Hill coefficient (*n*) and the ODCs provide a way to assess these effects:

(2) $${\text{SO}}_{2}=\frac{{\left(\text{PO}2\right)}^{n}}{{\left({\text{P}}^{50}\text{O}2\right)}^{n}+{\left(\text{PO}2\right)}^{n}}$$

where P^50^O_2_ is O_2_ half-saturation pressure of Hb.

**Figure 1:**
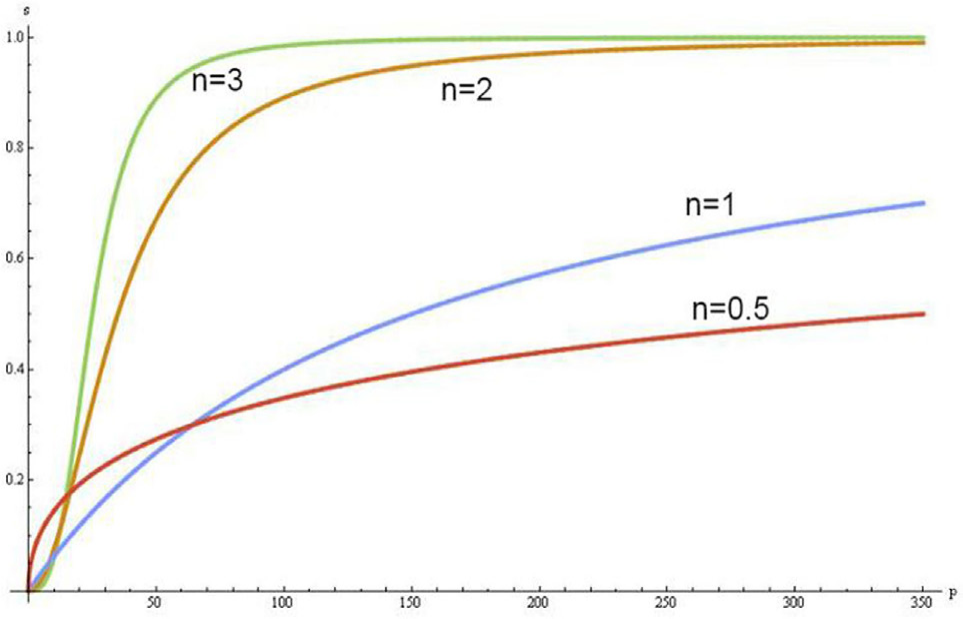
Oxyhemoglobin dissociation curves according to different Hill coefficients.

The increase of concentrations of the negative allosteric effectors in erythrocytes shifts the sigmoidal shape of ODC to the right and when Hb saturates with them, the ODC becomes hyperbolic in form (Figure 1). In this condition, Hb converts from the quaternary conformational state R (relax) to a state T (tense), which has a lower Hb–O_2_ affinity, hyperbolic form of ODC and increases up to six times the buffer capacity of Hb (24).

## Physiology and Pathophysiology

**Figure 2:**
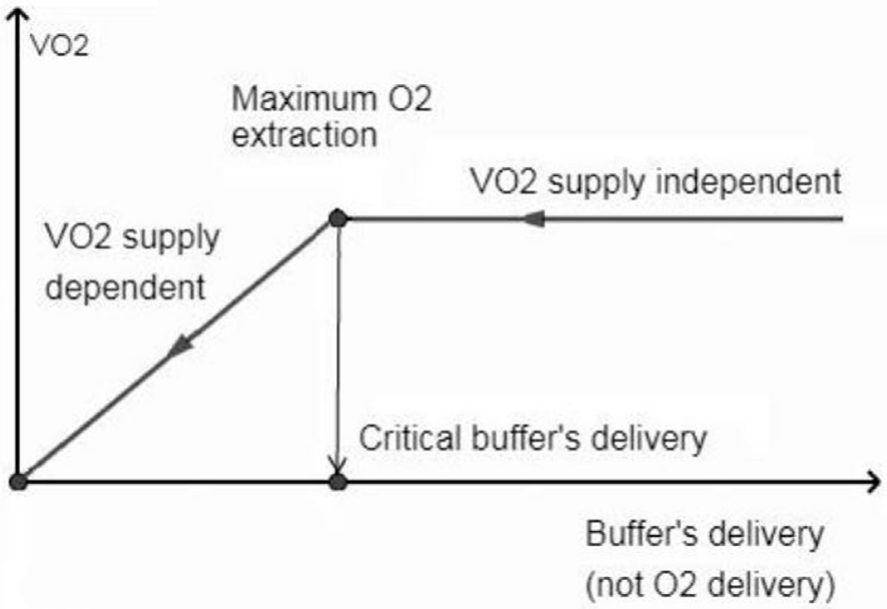
Oxygen consumption (VO_2_) is independent when perfusion allows hemoglobin not to be fully saturated with protons. From the critical buffer’s delivery or maximum O_2_ extraction point, the CO_2_ abruptly accumulates in the brain tissue, causing VO_2_ dependence on blood flow buffering capacity (not on O_2_ delivery) to counteract the life threatening acidosis.

In brain tissue (RQ at 1.0), use of 1 mol of O_2_ will result in the production of more CO_2_ than in the other tissues (RQ at 0.8). That is, the Hb buffering capacity is depleted with higher SO_2_ in the brain tissue (i.e., SO_2_ ≈40%, Eq. 1) than in other tissues. From this critical point, the generated CO_2_ is transported only in dissolved states and accumulated in the brain tissue causing clinically important acidosis with “normal” values of PtiO_2_. High levels of CO_2_ and intracellular acidosis directly or indirectly suppress the mitochondrial respiration: the CMRO_2_ is inhibited proportionally to decrease the perfusion by compensatory mechanisms [i.e., OEF is unable to raise (25)] trying to counteract the life-threatening intracellular acidosis. This is a blood-buffering capacity (BBC)-dependent mitochondrial suppression while independent of tissue O_2_ availability for cell aerobic metabolism. The O_2_ consumption (VO_2_)/buffer’s delivery inter-independency is shown in Figure 2.

Therefore, the accepted term “O_2_ delivery” does not reflect the pathophysiology of metabolism and has to be replaced by “buffer’s delivery,” which depends on the amount of Hb, the CBF, the total content of CO_2_ in arterial blood, and the Hb quaternary conformational state at the end of capillaries. The exaggerated increase in PtiO_2_ by NH in the VO_2_ supply-dependent phase has no metabolic effect due to the depletion of BBC. Moreover, augmentation in BBC in this phase through the increases of Hb concentration, augmentation of CBF or controlled hyperventilation without a change of Hb conformational state at the end of capillaries, is able to unlock the mitochondrial suppression and raise the CMRO_2_. Importantly, the high concentrations of 2,3 DPG as a significant negative allosteric effector can reduce the Hill coefficient to one without depleting the buffering capacity of Hb and can convert the ODC to hyperbola. In this case, the NH also causes an exaggerated increase in PtiO_2_ in relatively healthy tissue by physiological reduction on rCBF through vasoconstriction.

## Clinical Interpretation

Many drugs and techniques used commonly during therapy of severe TBI, including mannitol, thiopental, ketorolac, nimodipine, hypothermia, deep sedation, transfusion of red blood cells (RBC), etc., can reduce the PtiO_2_ in the damaged tissue (26–30). This is because it improves the relationship within BBC and CMRO_2_ causing a decrease of Hb loading by negative effectors (i.e., increase in Hb–O_2_ affinity). The effects of medically induced augmentation of cerebral perfusion pressure on cerebral oxygenation are difficult to predict (31, 32). Indeed, the effects depend on which phase (VO_2_ buffer supply dependent or independent) the rCBF (i.e., BBC) change is made. Coles et al. showed that with hyperventilation, many regions of brain demonstrate an increase in CMRO_2_ despite the reduction in CBF (33). In fact, the reduction of Hb loading by negative allosteric effectors in the VO_2_ buffer supply-dependent phase, the hyperventilation rehabilitates the suppressed mitochondrial function by augmentation of BBC. Often uncontrolled hyperventilation causes an important decrease in PtiO_2_ due to an increase in Hb–O_2_ affinity. The worst is, when hyperventilation causes end capillary T to R conformational change of Hb with a decrease in BBC, which is dangerous for damaged tissue. Unlike hyperventilation, hypoventilation improves tissue oxygenation (34). Despite this effect, hypoventilation has no clinical use in patients with TBI. The increase in PtiO_2_ is related not so much to the increase of CBF by vasodilatation as to the increase in Hb loading by the negative allosteric effectors and the decrease in Hb–O_2_ affinity. Depletion of BBC will cause the decrease of CMRO_2_. Several studies have shown an association between anemia and poor outcome after TBI (35–37). As expected, the higher Hb levels are associated with improved outcome after subarachnoid hemorrhage (38) and post-cardiac arrest patients (39–41) (i.e., higher BBC maintains the metabolism in the VO_2_ supply independent phase). On the other hand, the transfusion of RBC is an independent risk factor for poor neurological outcome after 3 and 6 months in TBI patients (42). The stored blood with low values of erythrocyte 2,3-DPG can be used without hesitation when correcting a chronic anemia for instance (43). However, in acute situations with important tissue acidosis, the organism needs to rapidly augment the buffering capacity of Hb by R to T states shift, but re-synthesis of 2,3-DPG is obviously insufficient in stored blood. In such situations, fresh blood or blood with a near normal 2,3-DPG content should be used (43). According to proposed hypothesis, the augmentation of BBC by increasing the Hb concentration is the main role of RBC transfusion in TBI patients. Inconsistent results and contradictory findings have been reported for the effects of NH in brain energy metabolism (8, 10, 19, 44–46). The lactate MR spectroscopy demonstrates that in ischemic core and contralateral striatum, the lactate levels are not affected by NH, whereas, in the region of mismatch, lactate levels changed with changes in O_2_ delivery (47). The T2*-weighted MRI studies showed positive CBF changes to O_2_ challenge in the mismatch region, in contrast to negative CBF changes in normal tissue due to O_2_-induced vasoconstriction (16, 48). Indeed, the increase in BBC through augmentation of rCBF in the VO_2_ buffer supply dependent phase (i.e., in mismatch region) improves local acidosis, unlock the mitochondrial suppression, and decrease the lactate levels.

## Discussion

Martini and colleagues determined that severe TBI management guided by PtiO_2_ monitoring was associated with a poor neurological outcome and was an inefficient use of hospital resources (49). Of course, recording of “normal” or high PtiO_2_ values with acceptable high inspired O_2_ fraction or positive end-expiratory pressures without sufficient BBC may create a false impression of safety and negatively impact clinical decision making. The use of absolute levels of PtiO_2_ as a marker of ischemia is confusing because a more important BBC deficit happens much earlier. The transition from the concept of “increase in O_2_ supply” to the concept of “increase of buffer supply” has important clinical implications in the strategy of treatment in neurotrauma patient. In addition, the characteristics of T Hb (i.e., hyperbolic ODC, high buffering capacity) allow us to understand and correctly assess the effects of current treatment and evolution of damaged brain tissue through PtiO_2_ changes with NH. The explanation of known paradoxes in neurotrauma such as an exaggerated rise in PtiO_2_ by NH, inexplicable decrease in PtiO_2_ after many drugs and techniques commonly used, and metabolic crisis without “ischemia” is explained for the first time by the Hb conformational changes and BBC-dependent mitochondrial suppression. As we have seen the metabolic crisis always coincides with ischemia but in the sense of lack of BBC. The hypothesis is structural and revolutionary. Despite the enormous amount of data available in literature, targeted practical observations are needed to confirm (or deny) this hypothesis.

## Conclusion

1. The exaggerated increase of PtiO_2_ by NH occurs in the “T” state of Hb when ODC as a hyperbole and does not always coincide with the mitochondrial dysfunction.
2. The BBC is a limiting factor of O_2_ consumption by damaged brain tissues.

## Author Contributions

GH, as the first author, he developed the hypothesis and prepared the manuscript. All the co-authors helped with their important input to finalize the hypothesis discussed and to write the manuscript.

## Conflict of Interest Statement

The authors declare that the research was conducted in the absence of any commercial or financial relationships that could be construed as a potential conflict of interest.
